# Epigenetic Control of Autophagy by MicroRNAs in Ovarian Cancer

**DOI:** 10.1155/2014/343542

**Published:** 2014-04-30

**Authors:** Rossella Titone, Federica Morani, Carlo Follo, Chiara Vidoni, Delia Mezzanzanica, Ciro Isidoro

**Affiliations:** ^1^Laboratory of Molecular Pathology, Department of Health Sciences, Centro di Biotecnologie per la Ricerca Medica Applicata, Università del Piemonte Orientale, Via P. Solaroli 17, 28100 Novara, Italy; ^2^Unit of Molecular Therapies, Department of Experimental Oncology and Molecular Medicine, Fondazione IRCCS Istituto Nazionale dei Tumori, 20133 Milan, Italy

## Abstract

Autophagy is a lysosomal-driven catabolic process that contributes to the preservation of cell homeostasis through the regular elimination of cellular damaged, aged, and redundant molecules and organelles. Autophagy plays dual opposite roles in cancer: on one hand it prevents carcinogenesis; on the other hand it confers an advantage to cancer cells to survive under prohibitive conditions. Autophagy has been implicated in ovarian cancer aggressiveness and in ovarian cancer cell chemoresistance and dormancy. Small noncoding microRNAs (miRNAs) regulate gene expression at posttranscriptional level, thus playing an important role in many aspects of cell pathophysiology, including cancerogenesis and cancer progression. Certain miRNAs have recently emerged as important epigenetic modulators of autophagy in cancer cells. The mRNA of several autophagy-related genes contains, in fact, the target sequence for miRNAs belonging to different families, with either oncosuppressive or oncogenic activities. MiRNA profiling studies have identified some miRNAs aberrantly expressed in ovarian cancer tissues that can impact autophagy. In addition, plasma and stroma cell-derived miRNAs in tumour-bearing patients can regulate the expression of relevant autophagy genes in cancer cells. The present review focuses on the potential implications of miRNAs regulating autophagy in ovarian cancer pathogenesis and progression.

## 1. Introduction

The research fields of autophagy and microRNAs (miRNAs) are relatively new (less than 20 years from their definition and discovery) and our knowledge of these fields is in tremendous expansion; on the other hand, the ovary cancer remains a deadly disease since no significant improvement in overall survival was achieved in the last three decades [1]. Here we focus on the involvement of macroautophagy in the pathogenesis of cancer and on the molecular significance of miRNAs that potentially regulate this process. Targeting of the autophagy pathway is being under evaluation as a new anticancer therapeutic option [2, 3]. Therefore, unravelling the clinical implications of autophagy-miRNA interaction in ovary cancer might hopefully open the way to new diagnostic and molecular therapeutic approaches for this highly malignant disease.

## 2. MicroRNAs and Cancer

Over the last decade, several classes of molecules that form a complex transcriptional regulatory network are being identified and still their complete characterization is ongoing [4]. The most well-known small noncoding RNAs, discovered nearly 20 years ago, are the miRNAs, which posttranscriptionally regulate gene expression through base pairing with the 3′-untranslated region of target mRNAs [5]. MiRNA-mediated repression of gene expression occurs through complex mechanisms not fully understood, including translational inhibition and mRNA degradation [6]. MiRNAs, as master regulators of gene expression, are among the major players in development, cell biology, and disease onset; in fact, it has been estimated that miRNAs can regulate the expression of more than half of protein-coding sequences in mammalian genomes. Accumulating evidence shows that miRNA expression is dysregulated in many types of cancer and that they can act either as oncogenes or tumour suppressors, depending on the cellular context and the expression of the miRNA targets in the particular tissue (reviewed in [7]). The effects of miRNA deregulation in cancer progression, diagnosis, and therapy have been extensively reviewed [8, 9].

## 3. Autophagy and Cancer

### 3.1. Morphological Aspects and Biochemical Regulation of Autophagy in Brief

Autophagy refers to a cellular process committed to the lysosomal degradation of self-constituents [10, 11]. Depending on the mechanism through which the substrate is delivered into the lysosome, autophagy is classified as macroautophagy, microautophagy, and chaperon-mediated autophagy [12–15]. However, macroautophagy (now simply referred to as autophagy) is the process mainly subjected to fluctuations to comply the needs for keeping cell homeostasis in response to stressful injuries. Autophagy, in fact, is the only pathway allowing the degradation of macromolecular aggregates, portion of cytoplasm, membranes, and entire organelles [16]. In this process, the autophagy substrate is sequestered within a newly formed vesicle (named autophagosome) that subsequently fuses with several endosomes and lysosomes to form autophagolysosomes (or autolysosomes) in which the autophagy substrate is fully degraded by the lysosomal acid hydrolases [17]. The substrates are selectively incorporated within the nascent autophagosomes through the intervention of proteins, such as p62/SQSTM1 (sequestosome), NBR1 (neighbour of BRCA1 gene 1), and Nix/BNIP3, that bridge the substrate and the membrane-bound LC3 [18–20]. LC3 (light chain 3; the mammalian orthologue of yeast atg8) derives from the posttranslational modification of MAP-LC3 (microtubule-associated protein-LC3) and is specifically associated with autophagosomal membranes [21]. The autophagosome originates from the nucleation and expansion of a preautophagosomal structure, a double-layered omega-shaped semicircle originating from the smooth endoplasmic reticulum [22]. Eventually, this structure closes up to form the autophagosome, which entraps the cargo. While being on formation, the lipidated isoform LC3-II is inserted onto the internal and external membranes of the autophagosome. The autophagosomes then move toward the microtubular organizing center, where they meet and fuse with the lysosomes [23]. The cargo is then completely degraded, along with the internal membrane of the autophagosome, within the acidic lumen of the autophagolysosome [24]. LC3-II present on the internal membrane of the autophagosome is also degraded, so that its consumption serves as readout of the autophagy flux [21]. Finally, the monomeric substrates are then pumped out in the cytosol for recycling purposes [25].

The autophagy pathway is controlled by a variety of signalling molecules [26, 27]. The ULK1 (Unc51-like kinase 1, the homolog of the yeast Atg1) kinase is believed to master the induction of autophagy [28]. Its function is under the control of two upstream kinases, AMPk and mTOR. Schematically, the class I PI3-k-AKT signalling pathway negatively impinges on autophagy through the activation of mTOR complex 1 (mTORC1), which inhibits the ULK1 complex, while the LKB-AMPk signalling pathway positively regulates autophagy through the inactivation of mTORC1 and the direct activation of ULK1 [29]. The activation of these pathways is influenced by intracellular and extracellular factors. The availability of nutrients (essentially, glucose and amino acids) and of growth factors activates the class I PI3k-AKT-mTORC1 pathway, thus repressing autophagy, whereas starvation strongly induces autophagy [30, 31]. On the other hand, energy depletion (i.e., shortage of ATP), oxidative stress, and DNA damage activate the LKB-AMPk pathway and therefore trigger autophagy [32–35]. The ULK1 complex signals to (also known as Vps34), which forms an active complex with Beclin-1 (also known as ATG6 or Vps30) [31]. This complex is recruited at the level of the preautophagosomal structure and locally produces PI3P (phosphatidyl -3-phosphate), the starting platform for the recruitments of membranes necessary for the biogenesis of the autophagosome [12].

### 3.2. The Pathophysiological Role of Autophagy in Cancer

The role of autophagy in cancer biology is not unequivocal. While basal (constitutive) autophagy prevents carcinogenesis through the constant elimination of damaged molecules and organelles that may increase the probability of oxidative stress mediated DNA mutation [36], induced autophagy can help cancer cells to face adverse situations such as the metabolic stress due to hypoxia and hyponutrition or the damaged provoked by anticancer treatments [37, 38]. In addition, the upregulation of autophagy may switch cancer cells into a dormant state, thus posing the basis for tumour relapse [39–41].

Many oncogenes and oncosuppressors regulate autophagy [42]. In general, oncogenes (e.g.,* AKT, BCL2*) tend to repress autophagy, though for some of them (e.g.,* RAS*) the final effect is cell context dependent [43–46]. It has been proposed that the abnormal expression of oncogenes favours the induction of prosurvival autophagy in cancer cells experiencing a metabolic stress. By contrast, oncosuppressors (e.g., PTEN, TSC1/TSC2, and DAPk) positively regulate autophagy and thus their lack reduces or abrogates the level of basal and inducible autophagy. Consistently, loss of function of the oncosuppressors Beclin-1 [47, 48] or PTEN [49, 50] predisposes to spontaneous cancers. The role of the oncosuppressor p53 in the regulation of autophagy in cancer cells appears ambiguous: while nuclear DNA-binding proficient p53 promotes the transcription of certain autophagy genes [51], p53 mutants that reside in the cytoplasm hamper autophagy [52, 53].

Besides, microenvironmental factors (hypoxia, pH, oxidative stress, nutrient availability, cytokines, hormones, and growth factors) and the physical-metabolic interaction of tumour cells with surrounding cells (inflammatory cells, fibroblasts) in the matrix greatly influence the actual level of autophagy in the cancer cells [54–56].

## 4. Ovarian Cancer Genesis and Progression: The Potential Role of MicroRNAs and of Autophagy

### 4.1. Involvement of Autophagy in the Pathogenesis of Ovarian Cancer

Based on the traditional view, ovarian tumours arise from subsequent metaplastic changes in the ovarian surface epithelium that lead to the development of four main histologic types: serous, endometrioid, mucinous, and clear cell (for a review see, [57]). More recently, the correlation of clinicopathological features with genetic studies has suggested a new paradigm for the pathogenesis and origin of epithelial ovarian cancer based on a dualistic model of carcinogenesis that classifies ovarian cancer in two types [58]. Type I tumours comprise low grade serous and endometrioid carcinomas, clear cells, and mucinous carcinomas which develop in a stepwise fashion from well-defined precursor lesions. They are indolent and relatively genetically stable, being characterized by a variety of somatic mutations or amplification/deletion of oncogenes or oncosuppressors including* K-RAS*,* B-RAF*, and* PTEN* [59, 60]. In contrast, type II tumours comprise high-grade serous and endometrioid carcinomas, malignant mixed mesodermal carcinomas, and undifferentiated carcinomas; they are rapidly growing and highly aggressive. Type II tumours are chromosomally unstable and express mutated* TP53* in more than 95% of the cases and* BRCA* inactivation in up to 50% of high-grade serous tumours (for a review see [61]). Besides these genetic abnormalities, also epigenetic alterations in the expression of critical genes may occur during cancer progression, and these changes are reflected in the signalling pathways that govern cell proliferation, cell migration, dormancy, and chemoresistance. At least 15 oncogenes and 16 oncosuppressor genes have been found deregulated in ovarian cancers because of genetic or epigenetic alterations [62–64]. Many of these oncogenes and oncosuppressors have also been involved in the regulation of autophagy [65]. Indeed, there is experimental evidence linking autophagy to ovarian cancer genesis. For instance, poorly differentiated and highly malignant ovarian cancer cells were shown to express very low level of the autophagosomal marker LC3, compared to benign hyperplastic tissues and borderline ovarian tumours [66]. The expression of the oncosuppressor* BECN 1*, which activates PI3k III-dependent autophagy (see above), was found downregulated in ovarian cancers, compared to benign lesions [66]. Also* DRAM* (damage-regulated autophagy regulator) 2, a p53-transcribed gene that positively regulates autophagy [67], was found to be expressed at very low level in aggressive ovarian tumours [68]. As many as 60 to 80% of both sporadic and familial ovarian cancers have been shown to bear mutations and deletions of the oncosuppressor* TP53 *gene [64, 69, 70]. Deletion of* TP53* could favour high level of basal autophagy [71], whereas DNA-binding deficient p53 mutants, which are found in human ovarian carcinomas [72], are unable to sequester BCL-2 or BCL-XL and indirectly could inhibit autophagy [53]. On the other hand, the hyperactivation of mTOR, which results in suppression of basal autophagy, was associated with a poor prognosis in ovarian carcinoma patients [73]. Taken together, it seems that ovarian carcinogenesis associates with insufficient autophagy. Another interesting gene linking autophagy and ovarian cancer is the aplasia ras-homolog member 1 (*ARH1*; also known as* DIRAS3*), which codes for a ras-homolog 26 kDa GTPase. The expression of* ARHI* correlates with prolonged progression-free survival and has been found downregulated in more than 60% of ovarian cancers [74, 75].* ARH1* is an imprinted oncosuppressor gene (one allele is inherited in a hypermethylated form), and therefore one single event (deletion, mutation, or epigenetic silencing) affecting the functioning allele is sufficient to cause the loss of function [76, 77]. ARH1 protein has recently been shown to modulate autophagy and dormancy in ovarian cancer cells [40]. It was shown that reactivation of* ARHI* by stromal factors could rescue dormant ovarian cancer cells through modulation of autophagy [40].

### 4.2. Modulation of Autophagy by MicroRNAs

Considering the implications of both miRNAs and autophagy in cancer-related processes and given the lack of current evidence linking these two rapidly growing fields of research, we prompted to review miRNAs regulating autophagy.

Recently, Jegga et al. used a system biology-based approach to define the complex regulatory and functional networks of genes controlling the autophagy-lysosomal pathway and found miR-130, miR-98, miR-124, miR-204, and miR-142 as putative posttranscriptional regulators of this pathway at various levels [78].

In principle, autophagy could be regulated by miRNAs targeting the mRNA of key molecules that indirectly induce or suppress autophagy, as, for instances, miR-504 that negatively regulates p53 [79] or miR-20b that negatively regulates the expression of HIF-1*α* [80] or any miRNA implicated in the regulation of the PI3k-(PTEN)-AKT-mTOR pathway as is, for instance, the case of miRNAs targeting PTEN [81]. More recently, miRNAs specifically targeting the mRNA of autophagy proteins are being identified [82]. For instance, members of the miR30 family can target Beclin-1, ATG2, ATG5, and ATG12 [83, 84]; miR-130a targets ATG2B [85]; mi-R181a-1 targets ATG5 [86, 87]; miR-290-295 targets ATG7 and ULK1 [88]; miRNA-17 and miR-119a-5p target ATG7 [89, 90]; miR376b targets ATG4 and BECLIN-1 [91]; miR-630 targets ATG12 [86]; and miR-519 targets Beclin-1, ATG10, and ATG16L1 [86].

Here, we will focus on those miRNAs that are either up- or downexpressed in ovarian cancers and that potentially regulate autophagy.

### 4.3. MicroRNAs Aberrantly Expressed in Ovarian Cancer

Comparative miRNAs expression profiling of ovarian cancer and normal ovary epithelium specimens has been performed in several laboratories and the readers can refer to some excellent comprehensive reviews [92, 93]. The laboratory of Carlo Croce first reported on the differential expression of some miRNAs between normal and cancer ovary epithelial tissues, showing an upregulation of miR-200a/b/c, miR141, miR-21, miR-203, and miR-205 and a downregulation of miR-199a, miR-140, miR-145, miR-222, and miR-125b1 [94]. In another study, miR-21 was found as the most upregulated and miR-125b as the most downregulated miRNA in ovary cancer versus normal ovary epithelium tissues [95]. However, a clear consensus on the diagnostic and prognostic value of a miRNA signature has not been reached yet. One study reported the complete downregulation of 44 miRNAs (including the oncosuppressive miR-15a, miR-34a, and miR-34b) and the upregulation of miR-182 in late-stage ovary cancers [96]. Another group found miR-199a, miR-214, and miR-200a as the ones most upregulated and miR-100 as the most downregulated miRNA in high-grade and late-stage ovary cancers [97]. Also miR-200a, miR-34a, and miR-449b were found downregulated in late-stage ovary cancers [98]. Late-stage ovary cancers are associated with the acquisition of chemotherapy resistance and metastasis formation, with the latter resulting from the phenotypic transformation known as epithelial-to-mesenchymal transition (EMT). A miRNA signature of the mesenchymal-like phenotype of epithelial ovary cancer was shown to include miR-141, miR-200, miR-29c, miR-101, miR-506, and miR-128 [99]. Further, the response to chemotherapeutics (e.g., Platinum) was found to be associated with a particular miRNA signature that includes let-7i [100], hsa-miR-27a, hsa-miR-23a, and miR-378 [98, 101].

In searching for the molecular pathways responsible for the metabolic and phenotypic alterations associated with a certain miRNA signature, it must be taken into account that one single miRNA can target the mRNA of multiple genes and that one single mRNA can have multiple target sequences for different miRNAs. Recently, another level of complexity in the global regulation of gene expression by miRNAs has emerged. It was in fact shown that the overexpression of certain miRNAs could indirectly regulate the level of other miRNAs in ovarian cancer cells [102].

### 4.4. Regulation of Autophagy by MicroRNA Aberrantly Expressed in Ovarian Cancer

As stated above, the modulation of autophagy by environmental stressful conditions (nutrient depletion, hypoxia, oxidative stress, and chemotherapeutic drugs) and/or by genetic and epigenetic hints may confer resistance to the chemotherapeutic treatments in cancer cells and may also favour the EMT and metastasization of cancer cells [103]. MiRNAs could contribute to the modulation of autophagy in these situations. For instance, the treatment with cisplatin could induce chemoresistance-promoting autophagy through the downregulation of certain miRNAs targeting ATG proteins or the pathways that control autophagy. As an example, miR-214 was shown to confer cisplatin resistance in ovarian cancer cells by targeting PTEN [97], and PTEN is known to positively regulate autophagy [104]. PTEN expression is posttranscriptionally regulated by a set of miRNAs [81, 97, 105]. In ovarian cancers, overexpression of miR-21 correlated with late stage and metastasis and significantly decreased the expression of PTEN [106].

We have made an “in silico” search of the* ATG* genes that are potential target candidates of the most relevant miRNAs found aberrantly expressed in ovary cancers. In Table 1 we report the results obtained using two algorithms for the prediction of microRNA gene targets, namely, the “TargetScanHuman” [107] and the miRanda [108] software. We have considered three different sets of miRNAs: in Tables 1(a) and 1(b) are reported the miRNAs that were found aberrantly expressed (either up- or downregulated with respect to the normal ovary epithelium) in ovarian cancers and that are possibly involved in ovarian tumorigenesis and progression; in Table 2 are reported the miRNAs that apparently play a role in chemoresistance; in Table 3 are reported the miRNAs that were found involved in the epithelial-to-mesenchymal transition of the phenotype. For clarity, in Table 1 we have separately described the* ATG* genes coding for ATG proteins (a) and the genes coding for signalling molecules that directly or indirectly control the induction and progression of autophagy. (b) In the tables, we also describe the function of the proteins coded by the genes predictably targeted by the miRNAs. In general, the two algorithms agreed in the identification of* ATG* target genes for most of the miRNAs of interest. The main discordances between miRanda and TargetScan were relative to the recognition of ATG14L as target of miR-21, miR-214, miR-140, miR-15a, and miR15b, and of ATG13 as a target of miR-15a and miR15b.

For some of these miRNAs the* ATG *gene target has been validated in tumours other than ovarian cancer. Although these data should be considered with caution due to the possible context and tissue specificity of miRNA regulation, we can assume that some available information can be applied also to ovarian cancer. For instance miR-101, reported to act as inhibitor of autophagy in breast cancer by targeting STMN1, RAB5A, and ATG4D mRNAs [109], has been found downregulated also in ovarian cancer compared to normal tissue, and its reexpression exerted tumour-suppressive effects in ovarian carcinogenesis [110]. Of note, stathmin overexpression showed a significant association with poor prognosis in ovarian cancer patients [111]. In keeping with the potential of miR-101 to regulate autophagy and ovarian cancer progression, it is to be mentioned that its target RAB5A was shown to be upregulated and to promote cell proliferation in ovarian cancer [112]. Also, miR-30a, which negatively regulates the expression of Beclin-1 in ovarian cancer cells [113], was found deregulated in stage I ovarian cancer patients together with other miRNAs; in particular, it was downregulated in samples from relapsing patients [114, 115]. This result is in line with possible involvement of miR-30a in autophagy-dependent chemoresistance in ovarian cancers.

## 5. Conclusion: Clinical Implications and Future Perspectives

Modulation of autophagy has a great impact on the carcinogenesis process. In fact, depending on whether it is considered at the precancerous or at the advanced stage, up- or downregulation of autophagy may elicit either tumour-promoting or tumour-suppressive effects [116, 117]. The actual level of ongoing autophagy in the tumour cells is dictated by genetic mutations but also influenced by the epigenetic regulation of gene expression [65, 103]. In the context of the intricate involvement of autophagy in cancer progression, emerging data point to the role of miRNAs as regulators of autophagy gene expression. The immediate and acute modulation of protein expression mediated by miRNAs plays a fundamental role in the adaptive response of the cell metabolism to environmental stresses such as nutrient shortage, hypoxia, and genotoxic stress. Autophagy is one of the main stress response pathways. Therefore, the modulation of ATG proteins and/or of signalling molecules that regulate autophagy by miRNAs finally impacts the capability of the cell to overcome the stress. This aspect is of particular relevance when considering the cytotoxic response of cancer cells to a chemotherapeutic drug. Chemosensitivity could be rescued by manipulating the level of miRNAs targeting autophagy. In fact, certain miRNAs can target both the autophagy and the apoptosis pathways and therefore can influence the cross-talk between these two processes and determine whether the cancer cell will resist or succumb to the toxic drug. For instance, miR-199a-5p was shown to increase chemoresistance by simultaneously promoting autophagy and suppressing apoptosis. By downregulating Beclin-1 expression, miR-30a and miR-376b downregulate not only autophagy but also apoptosis since the level of free antiapoptotic BCL-2 protein in the cell will increase. Thus, miRNAs can act as molecular switches to turn on or off either or both of the autophagy and apoptosis processes. These findings provide the rationale for designing novel therapeutic approaches combining the conventional anticancer drugs with miRNAs targeting the autophagy process.

Autophagy is clearly deregulated in ovarian cancer (reviewed in [65]), and here we have highlighted the possibility that the miRNAs aberrantly expressed in ovarian cancer could be involved in such deregulation.

The miRNA landscape of ovarian cancer is in rapid progress [118] and advance in detection and functional evaluation of miRNAs is expected to strongly contribute to unravelling the network of apoptosis and autophagy regulation in this complex disease. In the near future, studies ongoing in our and other laboratories will likely identify the miRNA signatures associated with autophagy in ovarian cancer, thus posing the basis for the possible harnessing of these miRNAs as therapeutic targets, as well as possible diagnostic-prognostic tools.

## Figures and Tables

**Table:** (a)

miRNAs (involved in ovarian cancer progression)	Predicted autophagy Target Genes	Function	Target prediction	
			miRanda	TargetScan
hsa-miR-141 hsa-miR 200a	*ATG7 *	A ubiquitin-activating (E1) enzyme homolog that activates both ATG8/LC3 and ATG12	Yes	Yes

hsa-miR-199a -5p	*ATG14L *	A component of the class III PtdIns 3-kinase complex	No	Yes
	*ATG4D *	Processing of MAP1-LC3	Yes	Yes
	*BECN1 *	BCL-2 interacting myosin/moesin-like coiled-coil protein 1, part of the class III PtdIns 3-kinase complex (activating macroautophagy)	Yes	Yes

hsa-miR-214	*ATG14L *	A component of the class III PtdIns 3-kinase complex	No	Yes

hsa-miR-182	*ATG7 *	A ubiquitin-activating (E1) enzyme homolog that activates both ATG8/LC3 and ATG12	Yes	Yes
	*ATG16L1 *	A component of the ATG12-ATG5-ATG16 complex for the formation of autophagosome	Yes	Yes
	*MAP1LC3B *	Microtubule-associated protein 1 light chain 3, precursor of LC3-II inserted in autophagosomal membranes	Yes	Yes

hsa-miR-140-5p	*ATG14L *	A component of the class III PtdIns 3-kinase complex	No	Yes

hsa-miR-125b	*UVRAG *	Interacting with Beclin-1 and Bif-1 (activation and stimulation of macroautophagy)	Yes	Yes

hsa-miR-34a	*ATG4B *	Processing of MAP1-LC3	Yes	Yes
	*ATG9A *	A transmembrane protein involved in lipid transport for phagophore expansion	Yes	Yes

hsa-let-7a	*ATG4B *	Processing of MAP1-LC3	Yes	Yes
	*ATG9A *	A transmembrane protein involved in lipid transport for phagophore expansion	Yes	Yes
	*ATG16L1 *	A component of the ATG12-ATG5-ATG16 complex for the formation of autophagosome	Yes	Yes

hsa-miR-15a hsa-miR-15b	*ATG13 *	A component of the ULK1 complex needed for ULK1 kinase activity	No	Yes
	*ATG9A *	A transmembrane protein involved in lipid transport for phagophore expansion	Yes	Yes
	*ATG14L *	A component of the class III PtdIns 3-kinase complex	No	Yes

hsa-miR-210	*ATG7 *	A ubiquitin-activating (E1) enzyme homolog that activates both ATG8/LC3 and ATG12	Yes	Yes

hsa-miR-449b	*ATG4B *	Processing of MAP1-LC3	Yes	Yes

**Table:** (b)

miRNAs (involved in ovarian cancer progression)	Predicted autophagy Target genes	Function	Target prediction	
			miRanda	TargetScan
hsa-miR-141 hsa-miR 200a	*PTEN *	Protein/lipid phosphatase that reduces the level of PIP3, thus limiting the activation of AKT	Yes	Yes
	*TSC1 *	Tuberose Sclerosis Complex component that negatively regulates mTOR	Yes	Yes

hsa-miR 200b hsa-miR 200c	*PTEN *	Protein/lipid phosphatase that reduces the level of PIP3, thus limiting the activation of AKT	Yes	Yes

hsa-miR 21	*TSC1 *	Tuberose Sclerosis Complex component that negatively regulates mTOR	Yes	Yes
	*BCL2 *	Interactor of Beclin-1 (represses autophagy) and of BAX (represses apoptosis)	Yes	Yes

hsa-miR-125b	*UVRAG *	Interacting with Beclin-1 and Bif-1 (activation and stimulation macroautophagy)	Yes	Yes
	*BCL2 *	Interactor of Beclin-1 (represses autophagy) and of BAX (represses apoptosis)	Yes	Yes

hsa-miR-101	*MTOR *	Mammalian target of rapamycin (kinase) component of MTORC1 (that inhibits autophagy) and of MTORC2 (that phosphorylates Akt)	No	Yes
	*RAB5A *	Endocytic vesicle associated ras-homolog GTPase (involved in autophagosome formation)	Yes	Yes

hsa-miR-31	*RAB1B *	Endocytic vesicle associated ras-homolog GTPase (involved in autophagosome formation)	Yes	Yes

hsa-miR-34a	*BCL2 *	Interactor of Beclin-1 (represses autophagy) and of BAX (represses apoptosis)	Yes	Yes

has-let-7a	*TSC1 *	Tuberose Sclerosis Complex component that negatively regulates mTOR	Yes	Yes

hsa-miR-15a hsa-miR-15b	*BCL2 *	Interactor of BECLIN 1 (represses autophagy) and of BAX (represses apoptosis)	Yes	Yes
	* TSC1 *	Tuberose Sclerosis Complex component that negatively regulates mTOR	Yes	Yes
	*FKBP1A *	An immunophilin that forms a complex with rapamycin and inhibits mTOR activity	Yes	Yes

hsa-miR-155	*PDK1 *	Kinase that phosphorylates AKT in Thr308	Yes	Yes
	*RPTOR *	Regulatory associated protein of mTOR (component of MTORC1)	No	Yes

hsa-miR-99a hsa-miR-100	*MTOR *	Mammalian target of rapamycin (kinase) component of MTORC1 (that inhibits autophagy) and of MTORC2 (that phosphorylates Akt)	No	Yes

hsa-miR-449b	*BCL2 *	Interactor of Beclin-1 (represses autophagy) and of BAX (represses apoptosis)	Yes	Yes

**Table 2:** Genes coding for proteins involved in the autophagy pathway identified as targets of microRNA involved in the cytotoxic response to cis-Platinum in ovarian cancer (miRanda release, August 2010; TargetScan release 6.2.).

miRNAs (involved in cis-Pt response)	Predicted gene(s) involved in autophagy	Function	Target prediction	
			miRanda	TargetScan
hsa-miR-27a	*PDK1 *	Kinase for the phosphorylation of AKT in Thr308	Yes	Yes
	*TSC1 *	Tuberose Sclerosis Complex component that negatively regulates mTOR	Yes	Yes

hsa-miR-23a	*UVRAG *	Interacts with Beclin-1 and Bif-1 (activation and stimulation macroautophagy)	Yes	Yes
	*ATG12 *	A ubiquitin-like protein that modifies (autophagosome expansion)	Yes	Yes
	*BCL2 *	Interactor of Beclin-1 (represses autophagy) and of Bax (represses apoptosis)	Yes	Yes
	*PTEN *	Protein/lipid phosphatase that reduces the level of PIP3, thus limiting the activation of AKT	Yes	Yes
	*TSC1 *	Tuberose Sclerosis Complex component that negatively regulates mTOR	Yes	Yes
	*RAPTOR *	Regulatory associated protein of mTOR (component of MTORC1)	No	Yes

hsa-miR-378	—			

hsa-let-7i	*ATG4B *	Processing of MAP1-LC3	Yes	Yes
	*ATG16L1 *	A component of the ATG12-ATG5-ATG16 complex for the formation of autophagosome	Yes	Yes
	*TSC1 *	Tuberose Sclerosis Complex component that negatively regulates mTOR	Yes	Yes

**Table 3:** Genes coding for proteins involved in the autophagy pathway identified as targets of the microRNA involved in the epithelial-to-mesenchymal transition process in ovarian cancer (miRanda release, August 2010; TargetScan release 6.2.).

miRNAs (involved in EMT)	Predicted gene(s) involved in autophagy	Function	Target prediction	
			miRanda	TargetScan
hsa-miR-141 hsa-miR 200a	*ATG7 *	A ubiquitin-activating (E1) enzyme homolog that activates both ATG8/LC3 and ATG12	Yes	Yes
	*PTEN *	Protein/lipid phosphatase that reduces the level of PIP3, thus limiting the activation of AKT	Yes	Yes
	*TSC1 *	Tuberose Sclerosis Complex component that negatively regulates mTOR	Yes	Yes

hsa-miR 29c	*ATG14L *	A component of the class III PtdIns 3-kinase complex	No	Yes
	*PTEN *	Protein/lipid phosphatase that reduces the level of PIP3, thus limiting the activation of AKT	Yes	Yes

hsa-miR-101	*MTOR *	Mammalian target of rapamycin (kinase) component of MTORC1 (that inhibits autophagy) and of MTORC2 (that phosphorylates Akt)	No	Yes
	*RAB5A *	Endocytic vesicle associated ras-homolog GTPase (involved in autophagosome formation)	Yes	Yes

hsa-miR-506	—			

hsa-miR-128	*PDK1 *	Kinase that phosphorylates AKT in Thr308	Yes	Yes
	*TSC1 *	Tuberose Sclerosis Complex component that negatively regulates mTOR	Yes	Yes
